# Antisense oligonucleotide therapy for *KCNT1* encephalopathy

**DOI:** 10.1172/jci.insight.146090

**Published:** 2022-12-08

**Authors:** Lisseth Estefania Burbano, Melody Li, Nikola Jancovski, Paymaan Jafar-Nejad, Kay Richards, Alicia Sedo, Armand Soriano, Ben Rollo, Linghan Jia, Elena V. Gazina, Sandra Piltz, Fatwa Adikusuma, Paul Q. Thomas, Helen Kopsidas, Frank Rigo, Christopher A. Reid, Snezana Maljevic, Steven Petrou

**Affiliations:** 1 The Florey Institute of Neuroscience and Mental Health, The University of Melbourne, Parkville, Victoria, Australia.; 2 Ionis Pharmaceuticals, Carlsbad, California, USA.; 3 School of Medicine, University of Adelaide, Adelaide, South Australia, Australia.; 4 South Australian Health and Medical Research Institute, Adelaide, South Australia, Australia.; 5 Praxis Precision Medicines, Cambridge, Massachusetts, USA.

**Keywords:** Genetics, Neuroscience, Epilepsy, Gene therapy, Mouse models

## Abstract

Developmental and epileptic encephalopathies (DEEs) are characterized by pharmaco-resistant seizures with concomitant intellectual disability. Epilepsy of infancy with migrating focal seizures (EIMFS) is one of the most severe of these syndromes. De novo variants in ion channels, including gain-of-function variants in *KCNT1*, which encodes for sodium activated potassium channel protein K_Na_1.1, have been found to play a major role in the etiology of EIMFS. Here, we test a potential precision therapeutic approach in *KCNT1*-associated DEE using a gene-silencing antisense oligonucleotide (ASO) approach. We generated a mouse model carrying the KCNT1 p.P924L pathogenic variant; only the homozygous animals presented with the frequent, debilitating seizures and developmental compromise that are seen in patients. After a single intracerebroventricular bolus injection of a *Kcnt1* gapmer ASO in symptomatic mice at postnatal day 40, seizure frequency was significantly reduced, behavioral abnormalities improved, and overall survival was extended compared with mice treated with a control ASO (nonhybridizing sequence). ASO administration at neonatal age was also well tolerated and effective in controlling seizures and extending the life span of treated animals. The data presented here provide proof of concept for ASO-based gene silencing as a promising therapeutic approach in *KCNT1*-associated epilepsies.

## Introduction

Epilepsy of infancy with migrating focal seizures (EIMFS) (previously known as malignant migrating partial seizures of infancy) is one of the most severe developmental and epileptic encephalopathy (DEE) syndromes (1). Pharmaco-resistant, nearly continuous, multifocal seizures begin to occur during the first 6 months of life and are accompanied by a marked developmental regression or stagnation (1, 2). In addition, major axial hypotonia, as well as pyramidal and extrapyramidal signs, become more apparent with the progressive development of athetotic movements and other movement disorders (2–4). Many of these patients also display microcephaly and strabismus (2–4). The prognosis of this condition is very poor, with most patients being nonverbal and nonambulatory (2, 4, 5). Importantly, this syndrome is associated with a high mortality rate (ranging from 17% to 33%) (1, 2, 5), which can occur as a consequence of prolonged status epilepticus and respiratory failure (2).

Pathogenic gene variants have been identified in *KCNT1* and account for up to 50% of the etiology of EIMFS (1, 3, 6–10). *KCNT1* encodes the sodium activated potassium channel protein K_Na_1.1, which mediates an outward rectifying K^+^ current related to the slow hyperpolarization that follows repetitive action potential firing (11–16). Functional studies have shown that *KCNT1* pathogenic variants associated with epilepsy result in an overall gain-of-function effect on the channel activity, increasing the current up to 22-fold compared with the wild-type channel (6, 11, 17). For a subset of these pathogenic variants, changes in voltage dependence or Na^+^ sensitivity contribute to the increase in current (18, 19). Gain-of-function variants in *KCNT1* have also been found in patients with other epileptic syndromes, including autosomal dominant nocturnal frontal lobe epilepsy, Ohtahara syndrome, and Lennox-Gastaut syndrome (3, 5, 7, 8, 20, 21). The antiarrhythmic drug quinidine has been shown to reduce the pathogenic currents produced by some of the mutant KCNT1 channels in vitro (11, 17, 22–25) but has had a limited clinical translation due to unwanted side effects (QTc prolongation and increased risk for arrhythmia), thereby resulting in a limited therapeutic window (25–37). To date, there is no effective therapy to treat *KCNT1*-associated epilepsies.

RNA-targeted therapies have recently received significant attention for the recurrent examples of preclinical and clinical success, as well as fast-tracked development (38, 39). Some of these approaches are proving to be disease modifying in the treatment of progressive neurological conditions (40, 41). Of the RNA-targeted therapies, which include antisense oligonucleotides (ASOs), short interfering RNA (siRNA), antagomirs, microRNA mimetics, and DNAzymes, ASOs have provided the most impactful clinical benefit (38, 41, 42). ASOs have emerged as a therapeutic option for orphan pediatric neurogenetic conditions, including spinal muscular atrophy (43) and Duchenne’s muscular dystrophy (44). ASOs are synthetic, single-stranded nucleic acids typically of 10 to 30 nucleotides in length that bind to a specific complementary mRNA target sequence and modulate the expression of a specific gene at the RNA level (42). This control is achieved by different molecular mechanisms, from the steric block of ribosomal activity to regulation of RNA splicing (39, 42). ASOs that harness endogenous RNase H1 mechanisms are commonly used to specifically reduce the expression of mRNAs. These ASOs are referred to as gapmer ASOs because they contain central block of deoxynucleotide monomers needed to induce the cleavage of target mRNA (39, 42). The major advantage of ASOs as a therapeutic approach is that the interaction with the target is considerably more specific than traditional small molecule–based therapeutics (42).

To investigate the therapeutic potential of reducing *KCNT1* expression, we evaluated the efficacy of a *Kcnt1* gapmer ASO in a homozygous mouse model of a *KCNT1*-DEE pathogenic variant. Mice homozygous for the *Kcnt1* variant p.P905L (L/L) display spontaneous seizures, abundant interictal activity in the electrocorticogram (ECoG), behavioral abnormalities, and early death. After a single intracerebroventricular (i.c.v.) bolus injection of *Kcnt1* gapmer ASO at postnatal day 40, L/L mice showed a marked knockdown of *Kcnt1* mRNA, resulting in an almost complete abolition of seizures, prolonged survival, and improved performance in behavioral tests. A dose of gapmer ASO that produced more than 90% *Kcnt1* mRNA knockdown to mimic exaggerated pharmacology in wild-type mice was well tolerated, suggesting minimal on-target liability. The preclinical evidence presented here provides proof of concept for ASO-based gene silencing as a therapeutic approach in *KCNT1* gain-of-function epilepsies.

## Results

### The Kcnt1 L/L model of KCNT1 encephalopathy.

**Figure 1:**
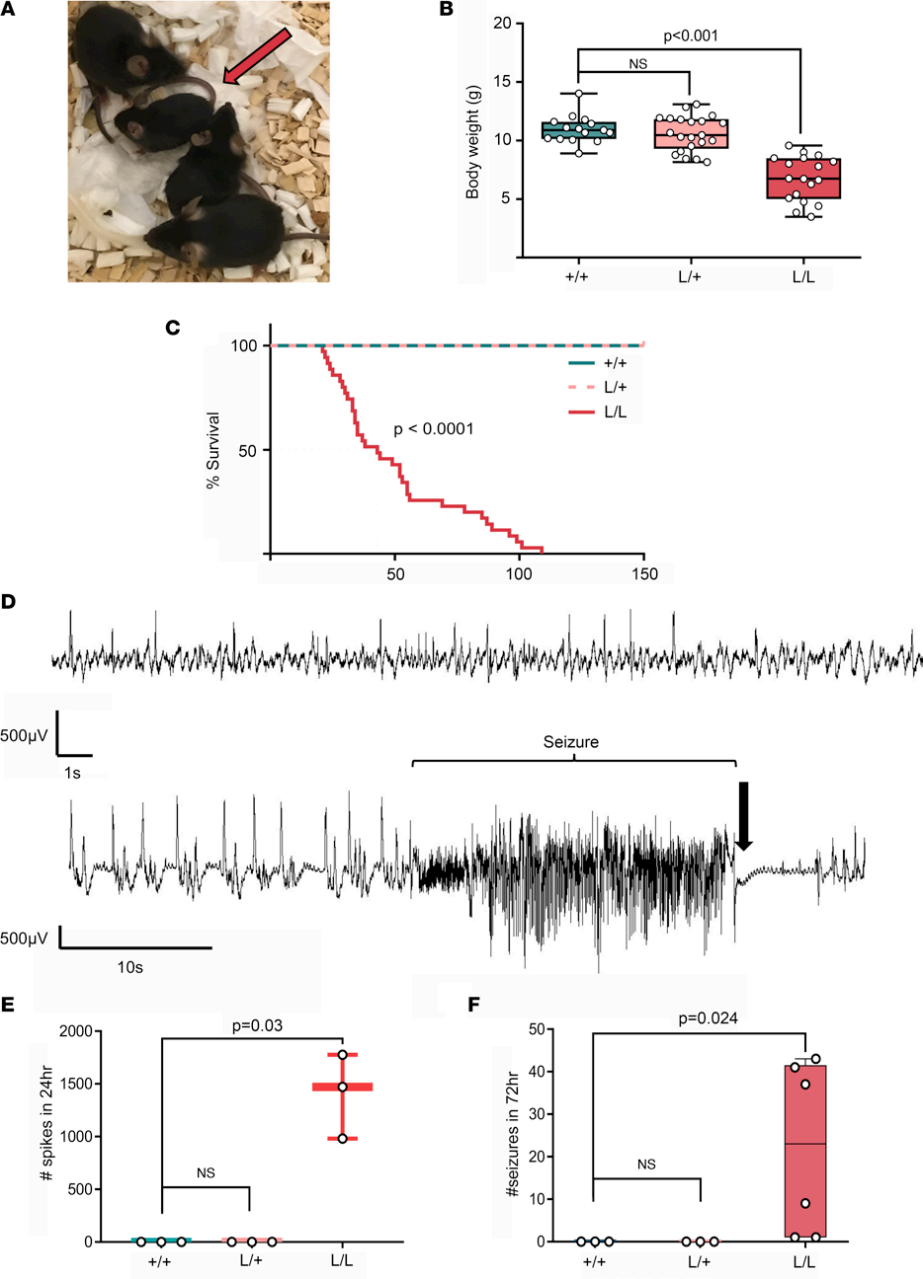
Phenotype of the *Kcnt1* L/L mouse model. (**A**) Difference in size at P21 of L/L mice (red arrow) compared with their L/+ and +/+ littermates. (**B**) Body weight is significantly reduced in L/L mice compared with their +/+ littermates at weaning age (Kruskal-Wallis test, followed by Dunn’s post hoc analysis; +/+ *n* = 15, L/+ *n* = 21, L/L *n* = 18). Data are presented in a box-and-whisker plot with maximal and minimal data points (whiskers) and median (line). (**C**) Life span is shortened in L/L mice with a median survival of 43 days (Kaplan-Meier curve, log-rank test *P* < 0.0001; +/+ *n* = 15, L/+ *n* = 15, and L/L *n* = 35). (**D**) Representative ECoG trace of ictal activity and interictal spikes in the L/L mice. Top: Acute interictal spikes. Bottom: Seizures can be preceded by an increase in frequency of acute interictal spikes. Spontaneous tonic-clonic seizures correlated with fast, high-amplitude signal, followed by electric suppression (black arrow). (**E**) Acute high-amplitude (>500 μV) spikes are present in the L/L mice, with a median of 1,470 spikes in 24 hours (*P* = 0.03, Kruskal-Wallis test, followed by Dunn’s post hoc analysis, *n* = 3 for each genotype). (**F**) Seizure frequency over 72 hours (+/+ *n* = 3, L/+ *n* = 3, L/L *n* = 6; median seizure frequency for L/L of 23 events. Kruskal-Wallis test, followed by Dunn’s post hoc analysis *P* = 0.024).

**Figure 2:**
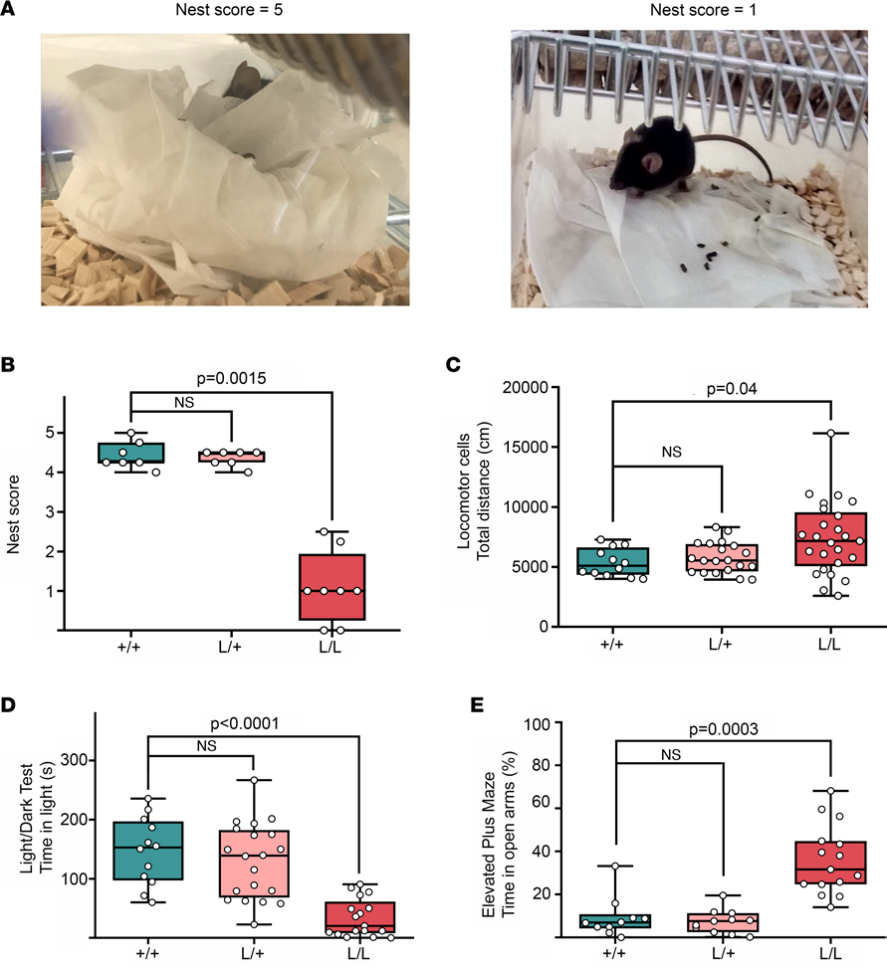
Behavioral profile of the *Kcnt1* L/L mouse model. (**A**) Representative images of minimal and maximal scores of nesting behavior. The left image corresponds to a score of 5 (+/+ mouse), while the right image exemplifies a score of 1 (L/L mouse). (**B**) Nesting behavior is impaired in L/L mice (+/+ *n* = 7, L/+ *n* = 7, L/L *n* = 8, Kruskal-Wallis test with Dunn’s post hoc analysis). (**C**) Total ambulatory distance explored in the locomotor cells test. L/L mice are more active compared with L/+ and +/+ (+/+ *n* = 12, L/+ *n* = 20, L/L *n* = 25, Kruskal-Wallis test with Dunn’s post hoc analysis). (**D**) L/L mice spend less time in the light compartment during the light/dark box test (+/+ *n* = 12, L/+ *n* = 20, L/L *n* = 17, Kruskal-Wallis test with Dunn’s post hoc analysis). (**E**) L/L mice display a preference for the open arms of the elevated plus maze (+/+ *n* = 10, L/+ *n* = 11, L/L *n* = 15, Kruskal-Wallis test with Dunn’s post hoc analysis). Data are presented in a box-and-whisker plot with maximal and minimal data points (whiskers) and median (line).

*Kcnt1* p.P905L heterozygous mice (L/+) showed no evident epileptic or behavioral phenotype (Figures 1 and 2) and no increased susceptibility to chemical or thermally induced seizures (Supplemental Figure 1, A–D; supplemental material available online with this article; https://doi.org/10.1172/jci.insight.146090DS1). In contrast, homozygous mice (L/L) were smaller (Figure 1, A and B) and had a markedly reduced life span compared with their littermates (median survival of 43 days) (Figure 1C). In addition, the yield of L/L mice, born from L/+ breeders, was not consistent with the predicted Mendelian ratio of 1:2:1 (+/+:L/+:L/L), with only 8.8% of pups born found to be L/L by age P8–P12 (age at which mice were sampled for genotyping) (Supplemental Figure 1E and Supplemental Table 1). Spontaneous tonic-clonic seizures were observed as early as P18. Video monitoring and handling showed diverse seizure phenotypes, with milder seizures lasting approximately 1 to 2 minutes and involving clonic movements of the forelimbs, neck, and head while in a seated posture (Supplemental Video 1), which were consistent with a Racine seizure intensity stage of 3 to 4 (45). More severe seizures included generalized tonic-clonic episodes with Straub tail, wild running and jumping, loss of postural tone, and tonic hind limb extension (Racine 5 and 6). Isolated atonic seizures (pure postural tone loss) were also observed. Milder seizures, in general, were not followed by an evident postictal state, and the mice quickly recovered (Supplemental Videos 2–4). Longer and generalized seizures were followed by a short period of complete immobility and rapid breathing with later episodes of reduced mobility lasting up to 10 minutes. Status epilepticus (a seizure lasting >5 minutes) was also observed and in most cases resulted in death. The frequency of tonic-clonic seizures was variable between mice (Figure 1F). ECoG recordings revealed the presence of frequent high-amplitude interictal acute spikes in the L/L mice (Figure 1D); this signal was absent in L/+ and +/+ (Figure 1E) and did not correlate with changes in behavior. Convulsive seizures were associated with ictal ECoG signals characterized by clusters of high-amplitude sharp-wave activity followed by electrical suppression at the end of the seizure (Figure 1D and Supplemental Video 5). No differences in seizure frequency or survival were observed based on sex (data not shown).

Behavioral abnormalities were also found in the L/L mice. Nest-building ability, an identified measure of general well-being in mice (46, 47), has been shown to be sensitive to brain lesions, the application of pharmacological agents, and the effects of genetic variants (48). In mouse models, instinctual nest-building performance is used to simultaneously assess general social behavior, cognitive performance, and motor performance (49). At P40, the nesting behavior in the L/L mice was markedly impaired compared with their +/+ and L/+ littermates (Figure 2, A and B).

Gross motor malfunction and exploratory behaviors were tested on the locomotor cell test and revealed a tendency toward hyperactivity, especially within the first 20 minutes of testing (Figure 2C). The light/dark box test showed L/L mice were prone to anxiety-like traits based on the limited time spent in the light chamber (Figure 2D). The elevated plus maze (EPM) was used to further explore anxiety-like traits. Interestingly, the L/L mice showed a marked preference for the open arms compared with their littermates (Figure 2E), suggesting a reduced fear of open and elevated spaces. Last, alterations in social behavior and cognition were tested in L/L mice using the 3-chamber social interaction test and Y maze test, respectively, but showed no significant difference compared with +/+ mice (Supplemental Figure 1, F and G).

### ASO-mediated Kcnt1 reduction rescues the seizure phenotype in L/L mice and improves overall behavior and survival.

**Figure 3:**
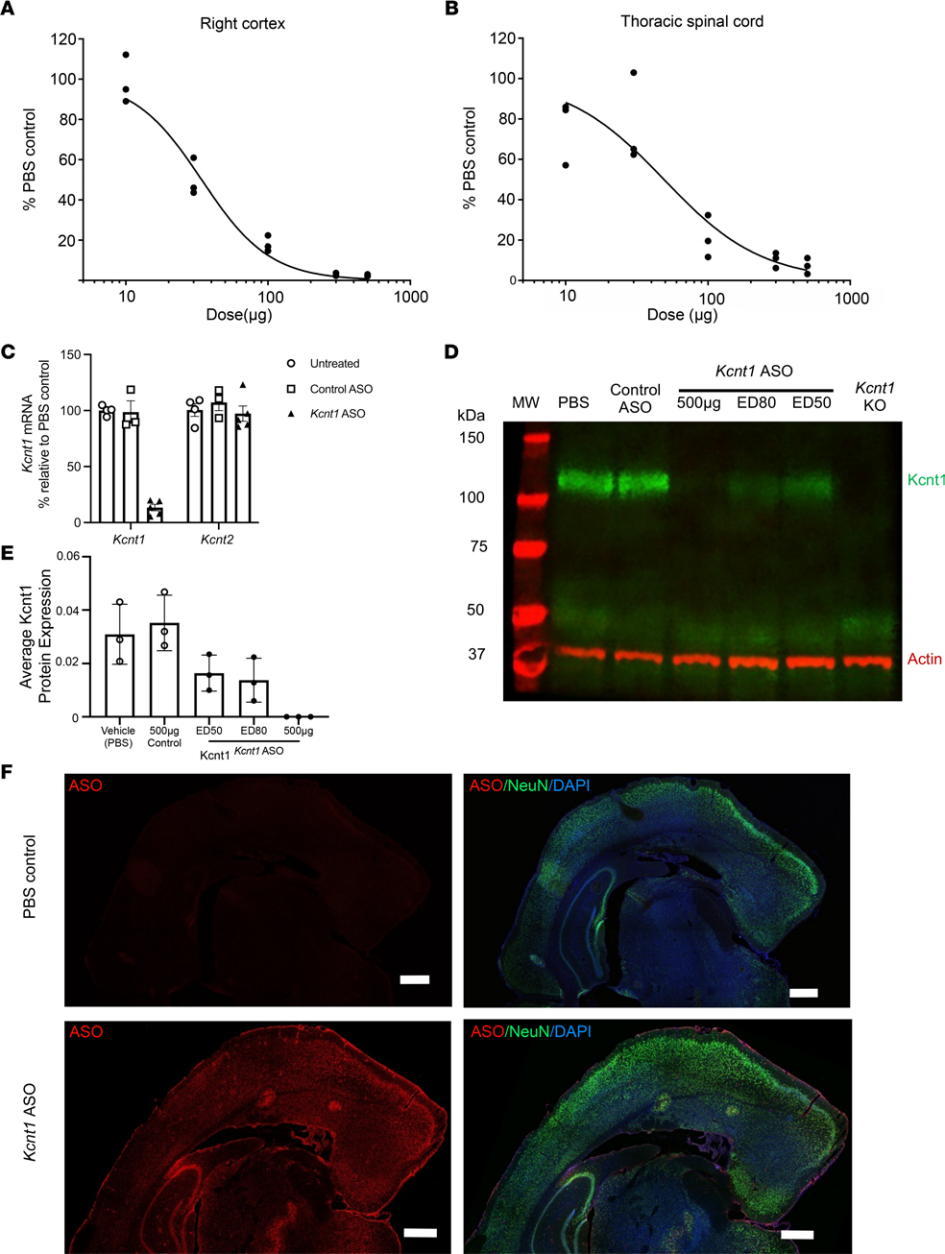
*Kcnt1* ASO produces a dose-dependent knockdown of *Kcnt1* mRNA in the mouse CNS. Dose-response curves for *Kcnt1* ASO in the brain cortex (**A**) and thoracic spinal cord (**B**) for +/+ mice injected at P40. The tissue was collected 2 weeks after injection and processed for mRNA quantification (*n* = 3 for each dose and PBS control, curves were fitted with the Motulsky regression). (**C**) Mouse cortex was collected 2 weeks after i.c.v. injection for mRNA quantification. The i.c.v. administration of *Kcnt1* ASO reduced the levels of *Kcnt1* mRNA (Kruskal-Wallis test *P* = 0.0027), without affecting the paralog gene *Kcnt2* (Kruskal-Wallis test *P* = 0.4794); data are presented as bar plots with mean and SEM, untreated *n* = 4, *Kcnt1* ASO *n* = 5, control ASO *n* = 3. (**D**) Western blot showing a reduction of Kcnt1 protein in WT mice left hemisphere 2 weeks after i.c.v. injection. (**E**) Average band signal for Kcnt1 protein. Data are presented as bar plots with mean and SD (*n* = 3 mice for each treatment condition, NS for all comparisons except PBS versus 500 μg, 1-way ANOVA with Dunnett’s multiple comparisons test). (**F**) Distribution of the *Kcnt1* ASO in the mouse brain. Coronal brain sections of +/+ mice treated with *Kcnt1* ASO 75 μg. Tissue was collected 2 weeks after i.c.v. injection and stained with an ASO antibody (red) and neuronal marker (NeuN; green) and counterstained with nuclear stain DAPI (blue). *Kcnt1* ASO was found throughout the meninges, hippocampus, and cortical layers (*n* = 3 experiments). Scale bars represent 500 μm.

To determine if the levels of *Kcnt1* mRNA could be reduced in a dose-dependent fashion, we tested a mouse-specific *Kcnt1* ASO in adult +/+ mice. At P40, *Kcnt1* ASO was delivered by i.c.v. injection with a range of doses (10, 30, 100, 300, and 500 μg), and vehicle-treated (PBS) animals received an injection of 10 μL of sterile Ca^2+^ and Mg^2+^ free PBS. Two weeks after treatment, the level of *Kcnt1* mRNA was determined using quantitative reverse transcription PCR (RT-qPCR) and compared with that of vehicle-treated (PBS) mice. The administration of *Kcnt1* ASO produced a dose-dependent knockdown of *Kcnt1* mRNA in the cortex and the spinal cord (Figure 3, A and B). We then determined the specificity of the *Kcnt1*-targeted knockdown by evaluating the expression of the highly homologous gene *Kcnt2* in +/+ mice treated with a bolus i.c.v. injection of 250 μg *Kcnt1* ASO and control ASO (nonhybridizing sequence). *Kcnt1* ASO produced a robust knockdown of *Kcnt1* mRNA in the cortex compared with untreated mice (Figure 3C) but did not affect the expression of *Kcnt2* mRNA (Figure 3D). No significant differences in *Kcnt1* gene expression were found between control ASO–treated and untreated mice, validating that the knockdown observed in *Kcnt1* ASO–treated mice resulted from on-target hybridization. A consistent reduction of the protein was observed 2 weeks after injection with ED_50_, ED_80_, and 500 μg of *Kcnt1* ASO (Figure 3, E and F). Using a pan-ASO antibody, which recognizes the phosphorothioate (PS) backbone of the ASO (50), we demonstrated broad ASO distribution in the mouse brain after a single bolus injection of 75 μg of *Kcnt1* ASO (Figure 3F).

### ASO-mediated Kcnt1 mRNA reduction rescues the seizure phenotype in L/L mice and improves overall behavior and survival.

**Figure 4:**
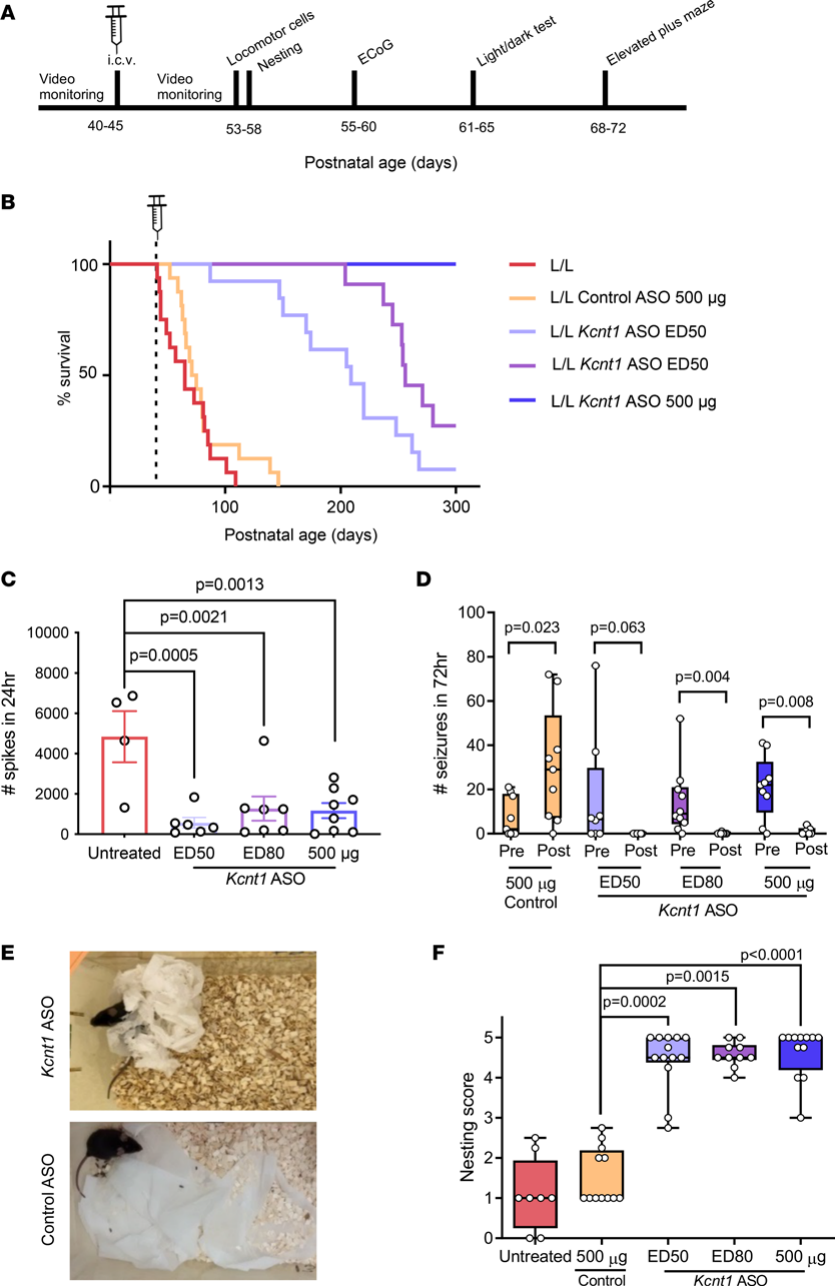
ASO-mediated knockdown of Kcnt1 at P40 markedly improves the disease phenotype of adult L/L mice. (**A**) Experimental timeline for behavioral studies. (**B**) Kaplan-Meier curves show a dose-dependent improvement in survival of adult L/L mice treated with Kcnt1 ASO (*P* < 0.0001 for Kcnt1 ASO ED_50_, ED_80_, and 500 μg, log-rank test), while mice treated with control ASO showed a survival similar to that of untreated animals (*P* = 0.237, log-rank test, untreated *n* = 16, control ASO *n* = 16, Kcnt1 ASO ED_50_ *n* = 13, ED_80_ *n* = 11, 500 μg *n* = 11). (**C**) Acute spike frequency over 24 hours (untreated *n* = 4, Kcnt1 ASO ED_50_ *n* = 6, ED_80_ *n* = 7, 500 μg *n* = 8; 1-way ANOVA *F*[3, 21] = 7.978, *P* = 0.001). (**D**) Seizure frequency was significantly reduced after treatment with Kcnt1 ASO ED_80_ and 500 μg. Although ED_50_ did not reach statistical significance, a trend toward reduction was observed. Treatment with control ASO did not reduce the occurrence of seizures (control ASO *n* = 9; ED_50_ *n* = 8; ED_80_ *n* = 10, 500 μg *n* = 9; seizure frequency was compared using the nonparametric Wilcoxon matched pairs signed-rank test, with Pratt’s method for identical rows). (**E**) Representative images of nesting behavior of Kcnt1 ASO ED_80_ (top) and control ASO–treated (bottom) L/L mice. (**F**) Nesting score of animals treated with Kcnt1 ASO showed a significant improvement compared with control ASO–treated animals (ED_50_ vs. control *P* = 0.0002; ED_80_ vs. control *P* = 0.0015; 500 μg vs. control *P* < 0.0001; untreated vs. control *P* > 0.9. Kruskal-Wallis test with Dunn’s post hoc analysis. Untreated *n* = 8, control ASO *n* = 12; ED_50_ *n* = 13; ED_80_ *n* = 10, 500 μg *n* = 12). Data are presented in a box-and-whisker plot with maximal and minimal data points (whiskers) and median (line).

Survival, seizure frequency, and behavioral markers (nesting, exploratory, anxiety like [light/dark box test and EPM]) were examined to determine the efficacy of *Kcnt1* knockdown (Figure 4A). Adult L/L mice were treated with a single bolus of *Kcnt1* ASO at ED_50_ (35 μg), ED_80_ (75 μg), or 500 μg. The control group received a dose of 500 μg of control ASO. Treating adult symptomatic L/L mice with a single dose of *Kcnt1* ASO resulted in an increase in survival. This effect was dose dependent and statistically significant across all the tested doses (median survival of 209 days for ED_50_, 256 for ED_80_, and >300 days for 500 μg *Kcnt1* ASO) (Figure 4B). In contrast, the animals treated with control ASO displayed a survival consistent with that of untreated L/L mice (median survival of 73 days for control ASO and 65 days for untreated mice). Mortality was often related to seizure and presented as status epilepticus.

L/L mice treated with a dose of ED_80_ and 500 μg of *Kcnt1* ASO had a significant reduction in the total seizure frequency; while the difference in the ED_50_ group was not statistically significant, a trend toward reduction was observed. In the group that received the control ASO, a significant increase in seizure frequency was seen (Figure 4D). Further, the presence of acute spikes was also reduced in *Kcnt1* ASO–treated animals 3 weeks after treatment (Figure 4C). Age-matched, untreated L/L mice were used as controls for ECoG, as the severity of the *Kcnt1* phenotype prohibited meaningful electrical recordings from being made in mice treated with control ASOs. During ECoG recording, none of the ASO-treated mice presented seizures, while there were 5 seizures recorded in the untreated group (representative traces of ECoG signal in treated and untreated animals are presented in Supplemental Figure 3). Together, these data indicate that the selective knockdown of *Kcnt1* mRNA has an anticonvulsant effect in our mouse model. In addition, mice treated with the *Kcnt1* ASO became less likely to exhibit seizures during routine handling, with no mice presenting seizures during the period of behavioral testing. In contrast, control ASO–treated mice continued to present seizures during routine handling.

Nesting behavior was evaluated 10–13 days after i.c.v. injection. At 48 hours after receiving new nesting material, an improvement in nesting behavior was noticed in all animals treated with *Kcnt1* ASO, independent of the dose received (Figure 4, E and F). Control-treated mice displayed poor nesting behavior, similar to what we observed in untreated L/L mice.

**Figure 5:**
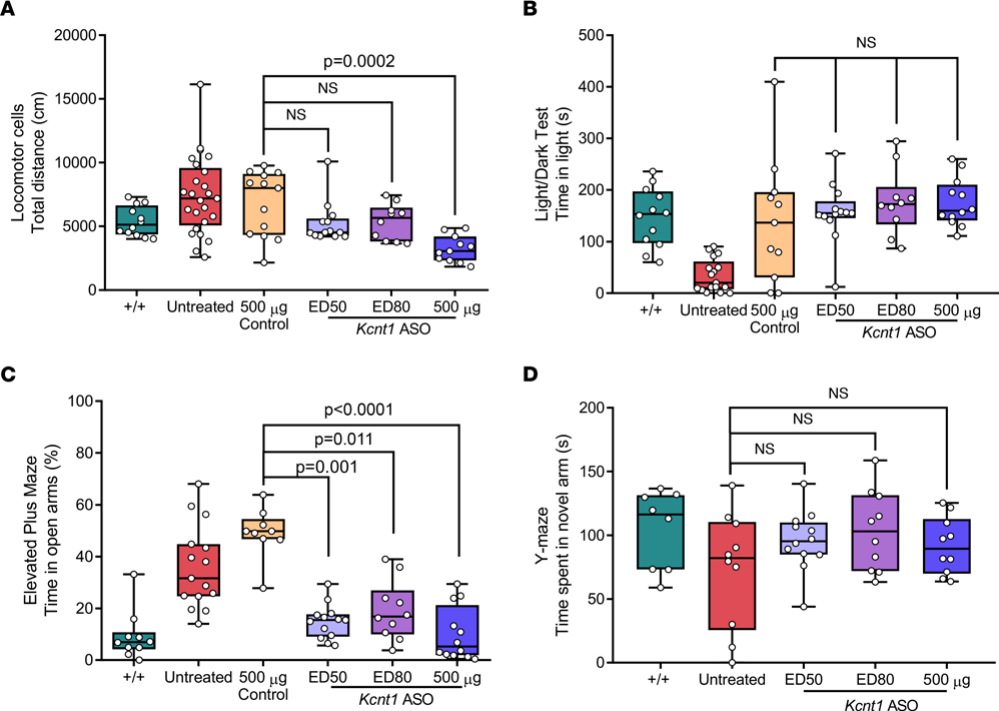
ASO-mediated knockdown of *Kcnt1* at P40 improves the behavioral phenotype of adult L/L mice. (**A**) Total ambulatory distance was reduced in mice treated with *Kcnt1* ASO 500 μg compared with control-treated mice (*P* = 0.0002) but not for mice treated with ED_50_ or ED_80_ (*P* = 0.775 and 0.839, respectively) (Kruskal-Wallis test, followed by Dunn’s multiple comparisons, +/+ *n* = 12, untreated *n* = 25, control ASO *n* = 13; ED_50_ *n* = 13; ED_80_ *n* = 10, 500 μg *n* = 11). (**B**) Time spent in the light compartment during the light/dark box test (Kruskal-Wallis test, followed by Dunn’s multiple comparisons *P* = 0.588; +/+ *n* = 12, L/L *n* = 17, control ASO *n* = 11, ED_50_ *n* = 13, ED_80_ *n* = 10, 500 μg *n* = 12). (**C**) Time spent in the open arms of the EPM (Kruskal-Wallis test, followed by Dunn’s post hoc analysis; +/+ *n* = 10; untreated *n* = 15; control ASO *n* = 9; ED_50_ *n* = 13; ED_80_ *n* = 10, 500 μg *n* = 12). (**D**) Time spent in the novel arm of the Y maze (Kruskal-Wallis test, followed by Dunn’s post hoc analysis *P* = 0.551; +/+ *n* = 8; untreated *n* = 10; ED_50_ *n* = 12; ED_80_ *n* = 10, 500 μg *n* = 10).

L/L mice treated with *Kcnt1* ASO ED_50_ and ED_80_ showed exploratory behavior similar to that of +/+, while mice that received 500 μg showed markedly reduced exploratory behavior (Figure 5A). In the light/dark box test, we found no difference in the time spent in the light compartment between mice treated with *Kcnt1* ASO and control ASO, showing an increase in this parameter for all mice that received an i.c.v. injection (Figure 5B). The EPM test showed that independent of the dose received, all *Kcnt1* ASO–treated mice spent less time in the open arms of the maze compared with control ASO–treated mice (Figure 5C). These data indicate that the ASO silencing of *Kcnt1* not only reduced seizure burden but also normalized behavioral markers of disease. Treatment with *Kcnt1* ASO did not alter the behavior of the L/L mice in the Y maze compared with untreated mice (Figure 5D).

### ASO-mediated Kcnt1 reduction improves general health and allows mating behavior, pregnancy, and parental behavior of L/L mice.

Due to the severity of the L/L mice phenotype, early mortality and behavioral abnormalities impaired normal mating behavior. To test if mating behavior could be restored, L/L mice received a single dose of 250 μg of *Kcnt1* ASO at P40. Two weeks after injection, male and female mice were set up as breeding pairs or trios. ASO-treated dams gave birth to litters of 5 to 8 homozygous pups. Although the first litter was often neglected, both male and female mice displayed effective parental behavior after the second pregnancy. The resulting L/L offspring displayed a phenotype as severe as the homozygous mice obtained from heterozygous breeding if left untreated.

### Neonatal administration of Kcnt1 ASO is efficacious and well tolerated.

**Figure 6:**
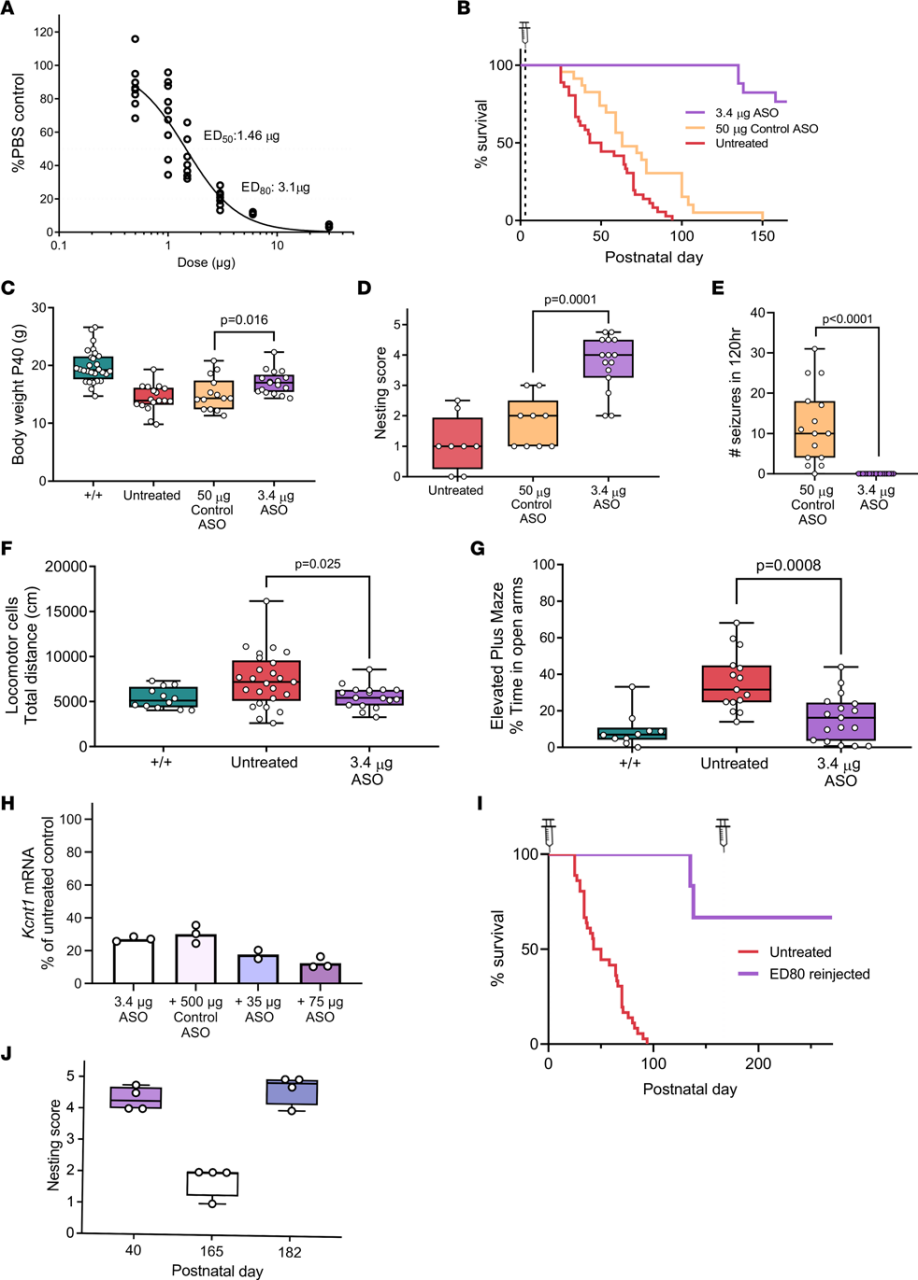
Neonatal administration of *Kcnt1* ASO in the L/L mouse model. (**A**) Dose-response curve for +/+ mice injected at P2 (*n* = 3–11 per dose). (**B**) Survival curve showing prolonged life span of *Kcnt1* ASO–treated mice (*P* < 0.0001). Control ASO produced a small improvement (*P* = 0.003, log-rank test) (untreated *n* = 36; control ASO *n* = 23; *Kcnt1* ASO 3.4 μg *n* = 17). (**C**) Weight at P40 (*P* = 0.016, +/+ *n* = 28, untreated *n* = 17, control ASO 50 μg *n* = 14, *Kcnt1* ASO 3.4 μg *n* = 17). (**D**) Nesting score (*P* = 0.0001, untreated *n* = 8; control ASO 50 μg *n* = 9; *Kcnt1* ASO 3.4 μg *n* = 15). (**E**) Seizure frequency (*P* < 0.0001, control ASO 50 μg *n* = 15, *Kcnt1* ASO 3.4 μg *n* = 17). (**F**) Ambulatory distance (+/+ *n* = 12, untreated *n* = 25, *Kcnt1* ASO 3.4 μg *n* = 17). (**G**) Time spent in open arms of EPM (+/+ *n* = 10, untreated *n* = 15, *Kcnt1* ASO 3.4 μg *n* = 17). Mann-Whitney test (**C**–**G**). Data are presented in a box-and-whisker plot with maximal and minimal data points (whiskers) and median (line). (**H**) Redosing at P30 further reduced *Kcnt1* mRNA (*Kcnt1* ASO 3.4 μg *n* = 3; *Kcnt1* ASO 3.4 μg + control ASO 500 μg *n* = 3; *Kcnt1* ASO 3.4 μg + *Kcnt1* ASO 35 μg *n* = 2; *Kcnt1* ASO 3.4 μg + *Kcnt1* ASO 75 μg *n* = 3). (**I**) Survival curve for L/L mice redosed with ED_80_ at P167 (untreated *n* = 36; ED_80_ reinjected *n* = 4). (**J**) Nesting score of L/L mice reinjected with ED_80_ (*n* = 4); *P* < 0.0001 (1-way ANOVA with Dunnett’s multiple comparisons test) for P40 versus P165.

To determine if the levels of *Kcnt1* mRNA could be reduced in a dose-dependent fashion in the neonatal period, we tested the *Kcnt1* ASO in newborn +/+ mice. At P2, the *Kcnt1* ASO was delivered by i.c.v. injection with a range of doses (0.5, 1, 1.5, 3, 6, and 30 μg), and vehicle-treated (PBS) animals received an injection of 2 μL of sterile Ca^2+^ and Mg^2+^ free PBS. Two weeks after treatment, the level of *Kcnt1* mRNA was determined and compared with that of vehicle-injected (PBS) mice. The early administration of *Kcnt1* ASO produced a dose-dependent knockdown of *Kcnt1* mRNA in the mouse cortex (Figure 6A).

Then we examined whether the early administration of *Kcnt1* ASO could prevent the development of a disease phenotype in the L/L model. At P2–P3, L/L mice received a single i.c.v. bolus injection of 3.4 μg (ED_80_), while control ASO mice received an injection of 50 μg. *Kcnt1* ASO–treated mice showed extended survival, with the first deaths being observed at P135 (Figure 6B) and displaying approximately 25% mortality by P150. Similarly, control ASO–treated mice also showed a short but significant improvement in survival (median survival of 63 days compared with 46.5 for untreated mice, Figure 6B). Seizure frequency improved significantly in mice treated with *Kcnt1* ASO (Figure 6E). Body weight was measured at P40 and was increased for *Kcnt1* ASO L/L mice compared with that of controls, but overall, body weight was still lower compared with +/+ mice (Figure 6C). Similar to adult treated L/L mice, behavioral markers including nesting and hyperactive exploratory activity (locomotor cells) and EPM showed improvement compared with untreated mice (Figure 6, D, F, and G).

Considering the likelihood that an ASO therapy for *KCNT1*-DEE in patients would require chronic ASO administration, we tested if a second dose was tolerated. A small group of +/+ mice received a 3.4 μg dose of *Kcnt1* ASO at P2 and a second injection of either *Kcnt1* ASO (35 or 75 μg) or control ASO (500 μg) at P30. The procedure was tolerated in all treatment groups. By P42, the administration of a second dose of 75 μg *Kcnt1* ASO showed a further reduction of *Kcnt1* mRNA compared with mice receiving control ASO on the second injection (Figure 6H).

In a small subset of L/L mice (*n* = 4) treated at P2, we tested if a second dose of *Kcnt1* ASO (75 μg) could be effective after the animals have become symptomatic (i.e., nesting behavior was deficient and seizures at handling were observed in 1 mouse). In these cases, a further extension in survival was observed (Figure 6I) and nesting behavior was rescued (Figure 6J).

### ASO-mediated Kcnt1 reduction in +/+ mice is well tolerated.

To evaluate the consequence of *Kcnt1* reduction, we treated adult +/+ mice with 500 μg *Kcnt1* ASO and performed an array of behavior tests. ASO-mediated knockdown of *Kcnt1* mRNA in +/+ mice did not affect their nesting behavior (Supplemental Figure 2A) and was consistent with a previously reported study on a *Kcnt1*-knockout (KO) mouse model. The exploratory behavior in the locomotor cell test was similar to that of control ASO–treated mice, and although the total ambulatory distance explored displayed a tendency toward reduced activity, this difference did not reach statistical significance (Supplemental Figure 2B). Similarly, a tendency to spend less time in the light chamber was observed (Supplemental Figure 2C).

On the EPM, +/+ *Kcnt1* ASO–treated mice showed a behavior similar to that observed in L/L *Kcnt1* ASO–treated mice, with a reduction in the time spent in the open arms (Supplemental Figure 2D). In the 3-chamber social interaction test, no difference was found between control and *Kcnt1* ASO–treated mice in the time spent with an intruder mouse (Supplemental Figure 2E). Finally, to further explore changes in memory, the mice were tested in the Y maze. A reduction in the median time spent in the novel arm was observed in *Kcnt1* ASO–treated mice compared with control (Supplemental Figure 2F).

## Discussion

In this study we investigated the potential of an ASO-based gene-silencing approach as a potential therapy for *KCNT1*-DEE. We have shown a gene-specific and dose-dependent knockdown of *Kcnt1* mRNA achieved with the *Kcnt1* ASO in L/L mice which significantly improved survival, epilepsy, and behavioral comorbidities and complete rescue of mating.

We first developed a rodent model and established disease biomarkers in homozygous animals that could be used for therapeutic screening. While heterozygosity for a gain-of-function variant in *KCNT1* is sufficient to result in a disease state in patients with gain-of-function *KCNT1* variants, this was not replicated in our mouse model. L/+ mice did not display an epileptic phenotype, increased susceptibility to seizures, or pathological behavioral changes. This is not completely unexpected, for mouse models of genetic epilepsy demonstrate considerable phenotypic variability based on factors including strain (51) and substrain (52) used. In contrast, homozygous mice presented a robust epilepsy phenotype, behavioral deficits, and a progressive, deteriorating course resulting in reduced survival, thus presenting a valuable tool for drug screening.

Recently, Shore et al. reported on a gain-of-function rodent model of KCNT1 epilepsy. This model was based on the KCNT1 p.Y796H variant, which has been identified as causing both inherited and de novo severe, early-onset autosomal dominant nocturnal frontal lobe epilepsy (ADNFLE) (25, 53). Patients with ADNFLE present with frequent, mostly nocturnal seizures and neuropsychiatric features (3, 7, 25, 27). Similar to our model, the *Kcnt1* p.Y777H heterozygous mice did not exhibit spontaneous epileptiform activity while the homozygous mice showed generalized tonic-clonic seizures and tonic seizures. In vivo electrocorticography in pups localized these seizures to the somatosensory cortex, and electrophysiology showed changes in membrane excitability with inhibitory neuron–specific impairments in action potential firing. Furthermore, the *Kcnt1* p.Y777H homozygous mice exhibited a hyperactivity phenotype in the open field and poor nesting behavior, similar to what we observed in our model. Interestingly, the p.Y777H model did not show alterations in the EPM, opposite to what we observed with our homozygous model.

Within 2 weeks of bolus administration, ED_80_ and 500 μg doses of *Kcnt1* ASO produced a significant reversal of the epileptic phenotype (Figure 4B), whereas dosing at ED_50_ showed only a trend toward seizure reduction, which could have been influenced by the sample size on this treatment group. A clear dose-dependent effect was observed for survival of adult L/L treated mice. Overall, the effectiveness of the ASO approach in controlling seizure activity verifies that brain hyperexcitability in this disease model is driven by *Kcnt1* gain of function, and therefore by reducing the excessive channel activity, the seizure threshold can be modified.

A critical concern shared among neurodevelopmental conditions and neurodegenerative disorders is whether disease progression can be stopped and the cognitive capacity preserved or improved once the pathological process has begun. Importantly, we have shown here that the downregulation of *Kcnt1* can be safe and effective in both neonatal (presymptomatic) and adult (symptomatic) mice not only in controlling spontaneous seizures but also in providing disease-modifying effects as indicated by improvements in cognition and behavior.

We also asked if the downregulatory ASOs had any harmful effects derived from excessive knockdown. Studies on *Kcnt1*-KO mice have reported some mild behavioral and cognitive alterations (54, 55). Here we show that the administration of a high dose of *Kcnt1* ASO to adult (500 μg) and neonatal (30 μg) +/+ mice did not result in serious adverse events. Treated adult mice showed a nesting behavior similar to that of untreated mice, consistent with the previously reported phenotype of *Kcnt1* KO (55).

Reduced exploratory behavior in the open field was reported in the *Kcnt1*-KO model characterization Bausch et al. published (54). Interestingly, while the initial exploratory activity of wild-type mice treated with *Kcnt1* ASO was similar to that of the control-treated group, we identified a trend that suggests an overall reduced exploratory activity. Similarly, in L/L mice treated with 500 μg of *Kcnt1* ASO, we observed a significant reduction in locomotor activity when compared with +/+. This behavior completely contrasted the hyperactivity observed in the untreated L/L mice and further supports that *Kcnt1* plays a role in the exploratory response to new environments. While seizures precluded us from testing the behavioral battery before and after treatment in our model, the overall improvement in survival and the observed improvement in behavior compared with untreated and ASO control–treated mice are consistent with significant improvement in cognition.

RNA-targeted ASO technology is an innovative therapeutic modality that has already achieved clinical success as an effective treatment for rare neurogenetic conditions like spinal muscular atrophy (43), Duchenne’s muscular dystrophy (44), and hereditary transthyretin amyloidosis (56). The FDA approval of nusinersen marked an important milestone for ASO technology. Nusinersen has demonstrated the disease-modifying properties and fulfilled the promise of precision medicine, opening a path for the further development of other RNA-based therapies for neurogenetic diseases (38). Although CNS-targeting ASOs require direct intrathecal delivery to be effective, this invasive approach is compensated by the extended half-life in the target tissue, and the wide brain distribution and high cellular uptake (57–60), which allows for less frequent dosing. In addition, direct delivery to the CSF compartment precludes the ASO from being distributed to the rest of the body, limiting the occurrence of adverse effects (57).

There is growing evidence that supports the development of ASO as a therapy for DEE. Successful use of ASOs for severe epilepsies has been reported in preclinical models of *SCN1A* (61), *SCN2A* (62), and *SCN8A* encephalopathy (63). Targeted augmentation of nuclear gene output (TANGO) ASO targets naturally occurring, nonproductive alternative splicing events to specifically reduce nonproductive mRNA and increase productive mRNA and protein of a target gene (64). Therefore, this approach can be used to address conditions with monogenic loss of function as a pathomechanism (64). Lim et al. showed that upregulation is not limited by the size of the gene and that the effect is titratable (64). Further, Han et al. have shown that the TANGO ASO modality can be used to upregulate the WT allele and compensate for the defective allele in a model of Dravet syndrome (*SCN1A* encephalopathy) (61). A significant reduction of the incidence of seizures and mortality related to sudden unexpected death in epilepsy were observed after a single i.c.v. administration of a TANGO ASO at P2 and P14 in a mouse model of Dravet syndrome (61). Furthermore, Lenk et al. have demonstrated the efficacy of a downregulating ASO targeting Scn8a in preclinical rodent models of both SCN8A and SCN1A DEE (63). Recently, Li et al. tested a PS gapmer ASO targeting *Scn2a* mRNA in a mouse model of early-onset *SCN2A* DEE. The Scn2a gapmer was shown to reduce spontaneous seizures, improve behavior (making it indistinguishable from wild-type animals), and significantly extend the life span of the treated mice, suggesting that ASO treatment can be effective and well tolerated (62).

Genetic neurodevelopmental conditions, including DEE, are devastating conditions that lack effective therapies. The unraveling of the genetic architecture of DEE has provided an exciting opportunity to develop precision medicines. Therapeutics that use RNA-targeting molecules are well positioned to directly address the underlying cause of DEE, including seizures and comorbid pathologies. In this study, we demonstrated that ASO-mediated reduction of *Kcnt1* is safe and has a disease-modifying effect in a homozygous mouse model of a *KCNT1* DEE pathogenic variant. These findings provide crucial evidence to support the development of ASO-based therapies for refractory epilepsies and developmental disorders that can contribute to reducing the impact of neurogenetic disorders.

## Methods

### Kcnt1 knockin mouse model

#### Mouse model generation.

Mice were housed in temperature-controlled rooms (≈22°C) on a 12-hour dark/12-hour light cycle, weaned at ≈P21, and maintained with rodent diet and water available ad libitum. Male and female mice were used for all experiments. Mice were housed in individually ventilated cages (IVCs, Tecniplast, Sealsafe plus mouse IVC green line) in groups of up to 5 animals. Mice that underwent ECoG electrode implantation, seizure monitoring, and nesting behavior assessment were housed individually during recovery from surgical procedures and for the duration of the test.

The L/L knockin mouse line was generated at the University of Adelaide using CRISPR/Cas9 to insert a single nucleotide variant at position c.2714, changing C>T. The *Kcnt1* p.P905L variant is homologous to the *KCNT1* p.P924L heterozygous pathogenic variant, which has been found in 2 patients with EIMFS (65, 66). *Kcnt1* p.P905L founders were generated as described previously (67). C57BL/6J zygotes were injected with 50 ng/μL of sgRNA (TGCATGAACCGCATGTTGGA), 100 ng/μL of *Cas9* mRNA, and 100 ng/μL of a single-stranded oligonucleotide repair template (Ultramer DNA from Integrated DNA Technologies). The oligonucleotide repair template sequence was GTTTGGAAAGAGCCAGAGAGTAGCTGTCCTTGGCACGGAACTGCATGAACCGCATGTTCGAAaGGTGTGTGAGCTCCGTGGTGATGCTGAGACTGGGGAAAAGCCTGAGGGAGGATGATCG.

Injected embryos were transferred to pseudopregnant females for further development. Pups were genotyped by PCR and the intended C>T variant was confirmed by Sanger sequencing. The colony was maintained on the C57BL/6J background and initially backcrossed to wild-type (+/+) mice to expand the colony with subsequent heterozygous intercross carried out to obtain wild-type (+/+), heterozygous (L/+), and homozygous (L/L) mice. Where possible *+/+* littermates were used as controls. When unavailable or as “intruders” for behavioral paradigms (specifically the 3-chamber social interaction test), C57BL/6J wild-type mice were purchased from the Animal Resources Centre (Australia).

For experiments with neonatal mice, C57BL/6J wild-type pregnant mice were purchased from the Animal Resources Centre. L/L litters were obtained from the crossing of *Kcnt1* ASO–treated L/L breeders.

#### Identification and genotyping.

Animals were toe-clipped at P8–P12 for identification, and the tip of the tail was biopsied for genotyping. DNA extraction was performed using the REDExtract-N-Amp Tissue PCR Kit (Merck Sigma).

The following PCR primers were used to amplify exon 24 of *Kcnt1*: Forward 5′-CCACCCAGTTATGACCACAG-3′ and Reverse: 5′-GCTGTAGGTATCTGTTAGCAG-3′.

PCR products were digested with the restriction enzyme BstBI (New England BioLabs), and the products were separated through electrophoresis on a 2% agarose gel stained with GelRed (Biotium). The wild-type allele generated a single fragment of 460 bp, and the mutant allele generated 2 fragments of 276 and 184 bp.

### Spontaneous behavioral seizures video recording

Mice older than P21 were housed individually in a 19.56 × 30.91 × 13.34 cm Thoren Caging System #9 small mouse II cage and monitored continuously for up to 5 days. Video recording was done using the Vivotek video server (VS8102) connected to an infrared day and night digital color camera (EVO2; Pacific Communications). Following recording, the videos were played back on a computer at 8×–13× speed using the VLC media player. Seizures that presented with any of the following behaviors were counted as an event: a) clonic seizure in a sitting position, b) clonic and/or tonic-clonic seizures while lying on the belly, c) pure tonic seizures, and d) clonic and/or tonic-clonic seizures while lying on the side and/or wild jumping. A tonic-clonic seizure lasting more than 3 minutes was considered to be prolonged, and a seizure with a duration of more than 5 minutes was considered to be a status epilepticus.

### Susceptibility to induced seizures

#### Thermogenic seizure assay.

Mice between the ages of P18 and P21 were placed in a container heated to constant 42°C for a maximum of 20 minutes. The latency to a first tonic-clonic seizure was recorded.

#### Pentylenetetrazol-induced seizures.

Mice between the ages of P30 and P40 were injected subcutaneously with the GABA antagonist pentylenetetrazol (80 mg/kg; Merck Sigma) dissolved in 0.9% sterile saline solution (Pfizer). The animals were then monitored for a maximum of 45 minutes, and latencies to a minimal (first tonic-clonic) and maximal (tonic hind limb extension) seizures were recorded.

#### Loxapine-induced seizures.

Mice between the ages of P30 and P40 received an intraperitoneal injection with the antipsychotic drug loxapine (100 mg/kg; Merck Sigma) dissolved in 0.9% sterile saline solution (Pfizer) and 25% DMSO (Merck Sigma). The animals were then monitored for a maximum of 1 hour, and the latency to a first tonic-clonic seizure was recorded.

In the 3 assays, animals were euthanized at the end of the experiment.

### Behavioral profile

Behavioral tests were conducted between 9 am and 6 pm, under similar lighting conditions for each task. The mice were transferred to the behavior testing room at least 30 minutes before the test. All mice were subjected to each behavioral test with a 5- to 7-day interval between each task.

#### Nesting behavior.

Two facial tissues (18 × 18.5 cm; 1.75–1.9 g) (Austwide Paper Products) were provided as nesting material 2 hours prior to the onset of the dark phase (5 to 6 pm). The quality of the nest was assessed 48 hours after. A score from 0 to 5 was given by adapting the scoring system of Hess et al. (46).

#### Locomotor cells.

Mice were placed in the center of a 27.31 × 27.31 × 20.32 cm covered chamber (Med Associates catalog ENV-510S-A) for 30 minutes, and their activity was monitored by a 48-channel infrared (IR) controller in 5-minute bins and analyzed with the Activity Monitor software version 7 (Med Associates).

#### Light/dark box.

The light/dark apparatus consisted of a 27.31 × 27.31 × 20.32 cm chamber (Med Associates catalog ENV-510S-A) divided into dark and light compartments of equal size by the insertion of a black Perspex box. The box contained a small opening in the middle, allowing the mouse to move between the compartments. The light chamber was brightly illuminated to 750 Lux by LED light lamps. Each mouse was placed in the center of the dark compartment and allowed to freely explore the box for 10 minutes. The latency to move into the light compartment as well as the amount of time spent in each side were automatically recorded and analyzed with Activity Monitor software version 7 (Med Associates).

#### EPM.

The EPM apparatus was made of light-colored Perspex and consisted of 2 open arms (5 × 30 cm) and 2 enclosed arms (5 × 30 × 14 cm) extending from a central area (5 × 5 cm). A raised lip (2.5 mm high, 5 mm wide) around the open arms was placed to reduce the likelihood of mice falling off the maze. The maze was elevated 60 cm above the floor. Each mouse was placed in the central area, facing an open arm. Their activity in the maze was video-recorded for 10 minutes. The time spent on each arm of the maze and in the center area was measured, and arm entries were counted using the TopScan software (CleverSys).

#### Three-chamber social interaction.

The test was conducted in a box (43 × 39 × 11 cm) made of transparent plastic and divided into 3 chambers; the middle section was 8 × 39 cm, and the 2 lateral sections were 16 × 39 cm each. The chambers were connected by rectangular openings in the middle. Metal mesh cages of 16 × 10 × 11 cm were placed in the 2 side chambers. The test consisted of 2 phases: habituation and trial phase. During the habituation period, the mouse was placed in the central chamber and allowed to explore freely all chambers for 10 minutes. After that, a preference for a chamber (left versus right) was assessed, and an unfamiliar mouse of similar age and size was placed in the metal cage of the side chamber of least preference. For the trial period, the mouse was again allowed to explore all chambers for 10 minutes. The activity during both habituation and trial period was video-recorded, and the amount of time spent in each chamber and near the cages was analyzed using TopScan software (CleverSys).

#### Y maze.

The Y maze was made of light-colored Perspex and consisted of three 7.5 × 30 × 14 cm arms separated by a 120° angle and containing a visual cue at the end. The test was performed in 2 phases: a training session and a trial session. During the training session the mouse was placed in the far end of one arm (home arm), facing away from other arms, and was allowed to explore this and one other arm (familiar arm) for 10 minutes. During this period, the third arm (novel arm) was not accessible. After a 1-hour interval, the mouse was again placed in the home arm but allowed to explore all 3 arms for 5 minutes (trial session). The test was video-recorded, and the amount of time spent in each arm was analyzed using TopScan software (CleverSys).

### Surgical procedures

#### ECoG electrode implantation surgery.

Mice were anesthetized with 3%–4 % isoflurane (IsoFlo; Abbott Laboratories) for induction and 2% for maintenance. A subcutaneous injection of meloxicam (1 mg/kg, ilium, Troy) dissolved in 0.9% saline was given prior surgery. Then, the mouse was placed in a stereotaxic apparatus (Kopf Instruments), after the scalp was shaved, sterilized with 80% ethanol, and infiltrated with lignocaine (1% ampules, Pfizer). A 1 cm incision was made on the scalp, and the skull was cleaned with 3% hydrogen peroxide solution (Sanofi). Four burr holes were drilled into the skull, and 3 screws were placed into the holes and used as epidural electrodes (catalog 8403, Pinnacle Technology) (1 reference electrode and 2 recording electrodes). A ball of silver wire was used as ground electrode. All electrodes were connected to a head mount (catalog 8201, Pinnacle Technology) and affixed to the skull with methyl methacrylate dental cement (catalog 1255710; Henry Schein; Lang Jet Denture Repair Acrylic). Then, mice recovered on a warming pad at 30 °C until fully awake.

#### ECoG recording and analysis.

Mice were allowed to recover for 3–5 days before recording. The mice were connected while awake to a mouse preamplifier (catalog 8406-SE) and an amplifier (catalog 8204 and 8206, Pinnacle Technology). Brain cortical activity was sampled on Sirenia (Pinnacle Technology) at 250 Hz for 25 hours; signal was band-pass-filtered at 0.5 to 40 Hz. ECoG signal was analyzed postacquisition using ClampFit 10.7 (Molecular Devices). A spike was identified when the amplitude was 2.5 times greater than the baseline activity and duration was shorter than 80 ms. An investigator blinded to the treatment groups/genotype reviewed and counted spike activity.

### Intracerebroventricular injections

#### Adult mice.

P40–P45 mice, weighing at least 10 g, were anesthetized using isoflurane (IsoFlo; Abbott Laboratories) at a concentration of 4%–5% mixed in O_2_ (vol/vol) for induction and 2% for maintenance. A subcutaneous injection of meloxicam (1 mg/kg) dissolved in 0.9% saline was given prior to surgery. The mice were positioned in a stereotaxic apparatus (myNeuroLab, Leica), and the scalp was cleaned with 80% ethanol. Then, the skin was infiltrated with lignocaine (1% ampules) and a 1 cm incision was performed. The skull was cleaned with 3% hydrogen peroxide solution to expose the bone sutures, and a burr hole was drilled at 0.8 mm lateral and 0.3 mm posterior to bregma. The tip of a 33G internal infusion cannula (Plastics One catalog C315I/SPC) was advanced to −3.0 mm from the skull surface to reach the right lateral ventricle. The cannula was connected to a 0.5 mL glass syringe (SDR) and an infusion pump (legato 210/210p syringe pump, Kd Scientific). A total volume of 10 μL of ASO or vehicle (sterile Ca^2+^ and Mg^2+^ free PBS) was delivered at a rate of 0.5 μL/s. One minute after completing the infusion, the cannula was withdrawn, and the skin was closed with 4–0 polyglactin 910 absorbable suture (coated Vicryl, Ethicon). Following surgery, the animals recovered on a Thermacage (Datesand, Ltd) until active and were then returned to their home cage.

#### Neonatal mice.

P2–P3 pups were cryo-anesthetized for 3 minutes. The scalp was cleaned with 80% ethanol and freehand injections were performed using a 32G needle Hamilton syringe (10 μL). Using lambda and the right eye as anatomical references, the needle was inserted midway between these 2 points and advanced –2.0 mm ventral from the skin surface (technique adapted from Kim et al., ref. 68). A total volume of 2 μL of ASO or vehicle (sterile Ca^2+^ and Mg^2+^ free PBS) was injected into the right lateral ventricle. Pups were gently warmed until skin color returned to pink and rolled over dirty bedding before being returned to their home cage. Maternal behavior was monitored for the following 10 minutes to ensure the pups were attended and returned to the nest.

### ASO synthesis

The *Kcnt1* ASO and control ASO (nonhybridizing sequence) were synthesized and screened in vitro by Ionis Pharmaceuticals as previously described (69, 70). Both molecules are 20 bp long and have the following structure: 5 modified nucleotides with 2′-MOE modifications at the 5′ and 3′ end and a central gap (gapmer) of 10 unmodified oligodeoxynucleotides. A PS backbone was used to enhance nuclease resistance. *Kcnt1* ASO targets the mRNA 3′ untranslated region at a position corresponding to 3981–4000 in transcript variant 1 (NM_175462.4).

The specific sequence for both ASOs is listed: *Kcnt1* ASO, 5′-GCTTCATGCCACTTTCCAGA-3′, and control ASO, 5′-CCTATAGGACTATCCAGGAA-3′.

ASOs were solubilized in sterile Ca^2+^ and Mg^2+^ free Dulbecco’s PBS (Merck Sigma), centrifuged (10,000 rotations per minute for 1–2 minutes at room temperature), and filtered through a 0.22 μm filter (Merck Sigma). A stock solution with a concentration of 50 mg/mL was stored at –20°C. Then, ASOs were further diluted to the desired concentration in sterile Ca^2+^ and Mg^2+^ free PBS (Merck Sigma) immediately before injection.

### Quantification of mRNA levels

#### Tissue harvest.

Two weeks after ASO administration, mice were deeply anesthetized with 4%–5% isoflurane, before being decapitated. After the brain was removed from the skull, the cerebellum and right and left cortex were dissected. The spinal cord was harvested using hydraulic extrusion as previously described (71), and a 1 cm piece from the low thoracic/lumbar cord was extracted. All dissected tissue was snap-frozen in liquid nitrogen, then stored at −80°C until RNA preparation.

#### Quantitative gene expression analysis.

Total RNA was isolated from the mouse right cortex and spinal cord using TRIzol reagent (catalog 15596026, Thermo Fisher Scientific) according to the manufacturer’s protocols. Contaminating genomic DNA was removed with recombinant DNAse I (rDNAse I) treatment (DNA-free Reagents; Ambion, Thermo Fisher Scientific). RNA was assayed for quality and quantity using a NanoDrop 2000c Spectrophotometer (Thermo Fisher Scientific).

#### RT-qPCR.

For RT-qPCR presented in Figure 3, A–D, oligo deoxythymidylic acid–primed cDNA was synthesized from 500 ng of total RNA using Murine Moloney Leukaemia Virus Reverse Transcriptase (Promega). RT-qPCR was performed on the ViiA 7 Real-Time PCR System (Applied Biosystems, Thermo Fisher Scientific) using SYBR Green technology and GoTaq qPCR master mix (Promega) according to the manufacturer’s protocols. The primers used for detection were *Kcnt1* Forward 5′-TCTTCCCTTTCTCAGGTCCAGG-3′ and Reverse 5′-AGGGAGAAGTTGAACAGCCG-3′, *Kcnt2* Forward: 5′-AAGGCTGAGCAGAAAAGGGC-3′ and Reverse: 5′-TCATTTCATCGTAGCCCACCG-3′, and *Rpl32* Forward: 5′-GAGGTGCTGCTGATGTGC-3′ and Reverse: 5′-GGCGTTGGGATTGGTGACT-3′.

Relative gene expression values were obtained by normalization to the reference gene Rpl32 using the −2ΔΔCt method, where −2ΔΔCt = ΔCt sample − ΔCt calibrator as previously described (72).

For Figure 6, A and H, and Supplemental Figure 3B, for RT-qPCR, 1 μg of rDNase I–treated, purified RNA was analyzed using a TaqMan RNA to Ct kit process (Applied Biosystems, Thermo Fisher Scientific) with a TaqMan probe to *Kcnt1* [probe ID Mm00558471_m1 (FAM-MGB)] in duplex with the mouse endogenous gene Glucuronidase Beta (*Gusb*) [probe ID Mm01197698_m1 (VIC-MGB)]. RT-qPCR was performed on the ViiA 7 Real-Time PCR System (Applied Biosystems) using cycling conditions specified by the manufacturer. All RT-qPCR reactions were performed in triplicate. Relative gene abundance values were calculated by normalization to *Gusb* and referenced to the control groups using the −2ΔΔCt method.

### Western blot

The quantification of Kcnt1 protein levels was performed by Western blotting. Total protein lysate was prepared from the mouse left hemisphere. The PVDF membrane was incubated with an antibody against Kcnt1 protein (anti-KCNT1 mse monoclonal [N3/26] 8 μg/mL, NeuroMab catalog 75-051), anti–β-actin (Mse IgG1 monoclonal dilute 1:10,000, Merck Sigma catalog A5441) as loading control, and IRDye IR secondary antibodies (IRDye 800CW, LI-COR catalog 926-32210, and IRDye 680RD, LI-COR catalog 926-68070).

### Immunohistochemistry

#### Tissue preparation.

Two weeks after ASO administration, mice were anesthetized with sodium pentobarbitone (80 mg/kg, lethal dose, i.p. injection) (ilium, Troy) and transcardially perfused with 0.1 M phosphate buffer (PB) followed by a 4% paraformaldehyde solution (pH 7.4). Brains were removed from the skull and stored in 20% sucrose solution overnight at 4°C, then frozen in isopentane cooled in liquid nitrogen before being sectioned coronally at 20 μm thickness with a cryostat (Leica Microsystems). Sections were mounted on Superfrost plus slides (Thermo Fisher Scientific) and stored at –20°C until used for immunostaining.

#### Immunostaining.

Sections were air-dried for 1 hour at room temperature and then blocked for 1 hour in a humidified chamber, with a mixture of 10% normal goat serum, 0.3% Triton X-100 (Merck Sigma) in PB. The blocked sections were incubated overnight with primary antibodies: polyclonal rabbit anti-ASO (against the PS backbone) 1:7,500 (Ionis Pharmaceuticals) and polyclonal guinea pig anti-NeuN 1:500 (Merck Sigma; catalog ABN90, clone A60 [MAB377]). After washing in PB, the sections were incubated for 2 hours with secondary antibodies: goat anti-rabbit Alexa Fluor 647 1:500 (Thermo Fisher Scientific; catalog A31573​) and donkey anti–guinea pig Alexa Fluor 488 1:500 (Thermo Fisher Scientific; catalog A-11073). To identify the nuclei, sections were then stained with DAPI (Merck Sigma). All incubations were conducted at room temperature. Slides were covered with ProLong Gold Antifade (Invitrogen, Thermo Fisher Scientific) and stored at –30°C. Confocal image stacks were acquired on a ZEISS LSM 780 microscope equipped with a 20×/0.8 NA lens. *Z*-stack images were acquired within Nyquist sampling parameters and deconvolved using Huygens 4.4.0 software (Scientific Software Imaging).

### Statistics

The latencies for the seizure susceptibility tests were plotted as Kaplan-Meier survival curves and tested for significance using the log-rank (Mantel-Cox) test. In all the survival plots, animals that did not reach the end point by 40 minutes (20 minutes for the thermogenic assay) were marked as censored. Dose-response curves were fitted with the Motulsky regression. For all other measures, Fisher test, Mann-Whitney *U* test, and nonparametric 1-way and 2-way ANOVA with post hoc analysis were used accordingly. Statistics were computed using GraphPad Prism. For all tests, statistical significance was set at *P* < 0.05.

### Study approval

All animal experiments were approved by the animal ethics committee of The Florey Institute of Neuroscience and Mental Health (protocols 14-026, 16-061, 16-062, and 17-014) and were performed in accordance with the guidelines of the National Health and Medical Research Council Code of Practice for the Care and Use of Animals for Experimental Purposes in Australia.

## Author contributions

LEB, ML, NJ, PJN, and S Petrou designed the research. LEB, ML, NJ, PJN, BR, A Sedo, A Soriano, KR, EVG, HK, and LJ performed the experiments. S Piltz, FA, and PQT designed and generated the *Kcnt1* mouse model. PJN, A Soriano, and FR contributed ASOs and provided advice on designing experiments. CAR, SM, and S Petrou supervised experimental design and provided advice on data interpretation. LEB, CAR, and SM wrote the manuscript. All authors read and contributed to the revision of manuscript.

## Supplementary Material

Supplemental data

Supplemental video 1

Supplemental video 2

Supplemental video 3

Supplemental video 4

Supplemental video 5

Supplemental video 6

## Version 1 09/29/2022

In-Press Preview

## Version 2 12/08/2022

Electronic publication

## References

[1] Burgess R (2019). The genetic landscape of epilepsy of infancy with migrating focal seizures. Ann Neurol.

[2] Coppola G (1995). Migrating partial seizures in infancy: a malignant disorder with developmental arrest. Epilepsia.

[3] https://www.ncbi.nlm.nih.gov/books/NBK525917/.

[4] McTague A (2013). Migrating partial seizures of infancy: expansion of the electroclinical, radiological and pathological disease spectrum. Brain.

[5] Borlot F (2020). KCNT1-related epilepsy: an international multicenter cohort of 27 pediatric cases. Epilepsia.

[6] Barcia G (2012). De novo gain-of-function KCNT1 channel mutations cause malignant migrating partial seizures of infancy. Nat Genet.

[7] McTague A (2018). Clinical and molecular characterization of *KCNT1*-related severe early-onset epilepsy. Neurology.

[8] Møller RS (2015). Mutations in KCNT1 cause a spectrum of focal epilepsies. Epilepsia.

[9] Kawasaki Y (2017). Three cases of KCNT1 mutations: malignant migrating partial seizures in infancy with massive systemic to pulmonary collateral arteries. J Pediatr.

[10] McTague A (2016). The genetic landscape of the epileptic encephalopathies of infancy and childhood. Lancet Neurol.

[11] Kaczmarek LK (2017). International Union of Basic and Clinical Pharmacology. C. Nomenclature and properties of calcium-activated and sodium-activated potassium channels. Pharmacol Rev.

[12] Kim GE, Kaczmarek LK (2014). Emerging role of the KCNT1 slack channel in intellectual disability. Front Cell Neurosci.

[13] Yuan A (2003). The sodium-activated potassium channel is encoded by a member of the Slo gene family. Neuron.

[14] Markham MR (2013). A sodium-activated potassium channel supports high-frequency firing and reduces energetic costs during rapid modulations of action potential amplitude. J Neurophysiol.

[15] Franceschetti S (2003). Na+-activated K+ current contributes to postexcitatory hyperpolarization in neocortical intrinsically bursting neurons. J Neurophysiol.

[16] Gao S (2008). Slack and Slick KNa channels are required for the depolarizing afterpotential of acutely isolated, medium diameter rat dorsal root ganglion neurons. Acta Pharmacol Sin.

[17] Milligan CJ (2014). KCNT1 gain of function in 2 epilepsy phenotypes is reversed by quinidine. Ann Neurol.

[18] Tang Q-Y (2016). Epilepsy-related slack channel mutants lead to channel over-activity by two different mechanisms. Cell Rep.

[19] Kim GE (2014). Human slack potassium channel mutations increase positive cooperativity between individual channels. Cell Rep.

[20] Lim CX (2016). KCNT1 mutations in seizure disorders: the phenotypic spectrum and functional effects. J Med Genet.

[21] Martin HC (2014). Clinical whole-genome sequencing in severe early-onset epilepsy reveals new genes and improves molecular diagnosis. Hum Mol Genet.

[22] Bhattacharjee A (2003). Slick (Slo2.1), a rapidly-gating sodium-activated potassium channel inhibited by ATP. J Neurosci.

[23] Yang B (2007). Slack and slick K(Na) channels regulate the accuracy of timing of auditory neurons. J Neurosci.

[24] Rizzo F (2016). Characterization of two de novoKCNT1 mutations in children with malignant migrating partial seizures in infancy. Mol Cell Neurosci.

[25] Mikati MA (2015). Quinidine in the treatment of KCNT1-positive epilepsies. Ann Neurol.

[26] Bearden D (2014). Targeted treatment of migrating partial seizures of infancy with quinidine. Ann Neurol.

[27] Fitzgerald MP (2019). Treatment responsiveness in KCNT1-related epilepsy. Neurotherapeutics.

[28] Chong PF (2016). Ineffective quinidine therapy in early onset epileptic encephalopathy with KCNT1 mutation. Ann Neurol.

[29] Fukuoka M (2017). Quinidine therapy for West syndrome with KCNTI mutation: a case report. Brain Dev.

[30] Jia Y (2019). Quinidine therapy for Lennox-Gastaut syndrome with KCNT1 mutation. A case report and literature review. Front Neurol.

[31] Madaan P (2018). A quinidine non responsive novel KCNT1 mutation in an Indian infant with epilepsy of infancy with migrating focal seizures. Brain Dev.

[32] Baumer FM, Sheehan M (2017). Quinidine-associated skin discoloration in KCNT1-associated pediatric epilepsy. Neurology.

[33] Abdelnour E (2018). Does age affect response to quinidine in patients with KCNT1 mutations? Report of three new cases and review of the literature. Seizure.

[34] Dilena R (2018). Early treatment with quinidine in 2 patients with epilepsy of infancy with migrating focal seizures (EIMFS) due to gain-of-function KCNT1 mutations: functional studies, clinical responses, and critical issues for personalized therapy. Neurotherapeutics.

[35] Numis AL (2018). Lack of response to quinidine in KCNT1-related neonatal epilepsy. Epilepsia.

[36] Yoshitomi S (2019). Quinidine therapy and therapeutic drug monitoring in four patients with KCNT1 mutations. Epileptic Disord.

[37] Mullen SA (2018). Precision therapy for epilepsy due to *KCNT1* mutations: a randomized trial of oral quinidine. Neurology.

[38] Levin AA (2019). Treating disease at the RNA level with oligonucleotides. N Engl J Med.

[39] Bennett CF (2019). Therapeutic antisense oligonucleotides are coming of age. Annu Rev Med.

[40] Muth CC (2018). ASO therapy: hope for genetic neurological diseases. JAMA.

[41] Rinaldi C, Wood MJA (2018). Antisense oligonucleotides: the next frontier for treatment of neurological disorders. Nat Rev Neurol.

[42] Crooke ST (2018). RNA-targeted therapeutics. Cell Metab.

[43] Finkel RS (2017). Nusinersen versus sham control in infantile-onset spinal muscular atrophy. N Engl J Med.

[44] Khan N (2019). Eteplirsen treatment attenuates respiratory decline in ambulatory and non-ambulatory patients with Duchenne muscular dystrophy. J Neuromuscul Dis.

[45] Lüttjohann A (2009). A revised Racine’s scale for PTZ-induced seizures in rats. Physiol Behav.

[46] Hess SE (2008). Home improvement: C57BL/6J mice given more naturalistic nesting materials build better nests. J Am Assoc Lab Anim Sci.

[47] Gaskill BN (2013). Nest building as an indicator of health and welfare in laboratory mice. J Vis Exp.

[48] Deacon RMJ (2006). Assessing nest building in mice. Nat Protoc.

[49] DeVos SL (2017). Tau reduction prevents neuronal loss and reverses pathological tau deposition and seeding in mice with tauopathy. Sci Transl Med.

[50] Kordasiewicz HB (2012). Sustained therapeutic reversal of Huntington’s disease by transient repression of huntingtin synthesis. Neuron.

[52] Kang SK (2019). C57BL/6J and C57BL/6N substrains differentially influence phenotype severity in the *Scn1a*
^+/-^ mouse model of Dravet syndrome. Epilepsia Open.

[53] Heron SE (2012). Missense mutations in the sodium-gated potassium channel gene KCNT1 cause severe autosomal dominant nocturnal frontal lobe epilepsy. Nat Genet.

[54] Bausch AE (2015). The sodium-activated potassium channel Slack is required for optimal cognitive flexibility in mice. Learn Mem.

[55] Bausch AE (2018). Loss of sodium-activated potassium channel slack and FMRP differentially affect social behavior in mice. Neuroscience.

[56] Mathew V, Wang AK (2019). Inotersen: new promise for the treatment of hereditary transthyretin amyloidosis. Drug Des Devel Ther.

[57] Bishop KM (2017). Progress and promise of antisense oligonucleotide therapeutics for central nervous system diseases. Neuropharmacology.

[58] Geary RS (2015). Pharmacokinetics, biodistribution and cell uptake of antisense oligonucleotides. Adv Drug Deliv Rev.

[59] Butler M (2005). Spinal distribution and metabolism of 2’-O-(2-methoxyethyl)-modified oligonucleotides after intrathecal administration in rats. Neuroscience.

[60] De Vivo DC (2019). Nusinersen in infants who initiate treatment in a presymptomatic stage of spinal muscular atrophy (sma): interim efficacy and safety results from the phase 2 NURTURE study (S25.001). Neurology.

[61] Han Z (2020). Antisense oligonucleotides increase Scn1a expression and reduce seizures and SUDEP incidence in a mouse model of Dravet syndrome. Sci Transl Med.

[62] Li M (2021). Antisense oligonucleotide therapy reduces seizures and extends life span in an SCN2A gain-of-function epilepsy model. J Clin Invest.

[63] Lenk GM (2020). Scn8a antisense oligonucleotide is protective in mouse models of SCN8A encephalopathy and Dravet syndrome. Ann Neurol.

[64] Lim KH (2020). Antisense oligonucleotide modulation of non-productive alternative splicing upregulates gene expression. Nat Commun.

[65] Epi4K Consortium (2013). De novo mutations in epileptic encephalopathies. Nature.

[66] Ohba C (2015). De novo KCNT1 mutations in early-onset epileptic encephalopathy. Epilepsia.

[67] Dawson RE (2020). Functional screening of GATOR1 complex variants reveals a role for mTORC1 deregulation in FCD and focal epilepsy. Neurobiol Dis.

[68] Kim J-Y (2014). Intracerebroventricular viral injection of the neonatal mouse brain for persistent and widespread neuronal transduction. J Vis Exp.

[69] McKay RA (1999). Characterization of a potent and specific class of antisense oligonucleotide inhibitor of human protein kinase C-alpha expression. J Biol Chem.

[70] Cheruvallath ZS (2003). Solid phase synthesis of phosphorothioate oligonucleotides utilizing diethyldithiocarbonate disulfide (DDD) as an efficient sulfur transfer reagent. Nucleosides Nucleotides Nucleic Acids.

[71] Richner M (2017). Hydraulic extrusion of the spinal cord and isolation of dorsal root ganglia in rodents. J Vis Exp.

[72] Livak KJ, Schmittgen TD (2001). Analysis of relative gene expression data using real-time quantitative PCR and the 2(−Delta Delta C(T)) method. Methods.

