# Supplementary material for: Antisense oligonucleotide therapy for KCNT1 encephalopathy
Source: JCI Insight. 2022 Dec 8;7(23):e146090. doi: 10.1172/jci.insight.146090 (PMC9746904; doi:10.1172/jci.insight.146090)
Supplement: Supplemental data [file jciinsight-7-146090-s071.pdf]

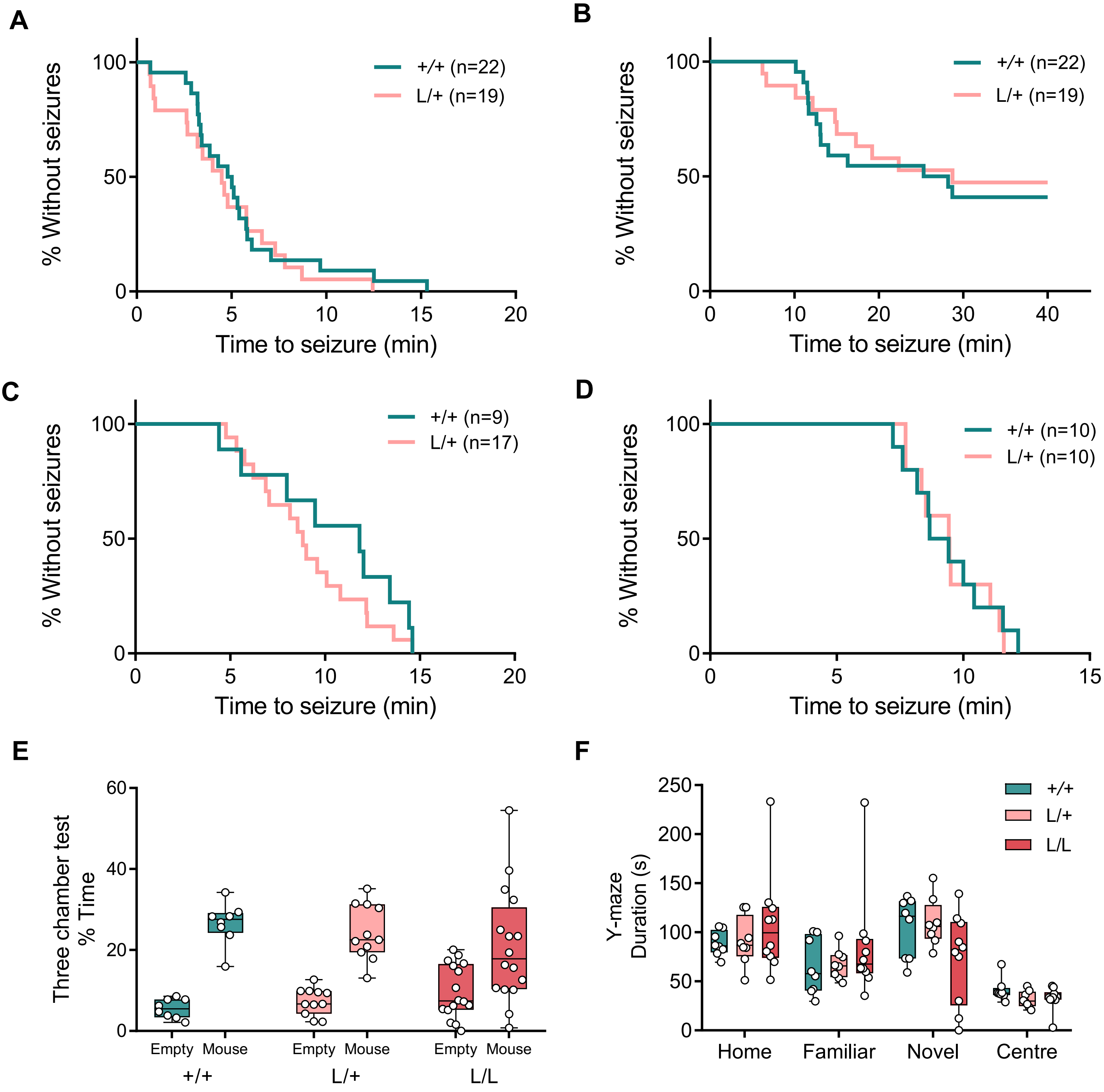
A
% Without seizures
+/+ (n=22)
L/+ (n=19)
Time to seizure (min)
B
% Without seizures
+/+ (n=22)
L/+ (n=19)
Time to seizure (min)
C
% Without seizures
+/+ (n=9)
L/+ (n=17)
Time to seizure (min)
D
% Without seizures
+/+ (n=10)
L/+ (n=10)
Time to seizure (min)
E
Three chamber test
% Time
Empty
Mouse
+/+
L/+
L/L
F
Y-maze
Duration (s)
+/+
L/+
L/L
Home
Familiar
Novel
Centre

Supplemental Figure 1. Extended phenotypic characterization of the *Kcnt1* p.P905L mouse model. Chemo-convulsant challenge with PTZ showed no increased susceptibility in L/+ mice for a first tonic-clonic seizure (**A**) (Kaplan-Meier curve, Log-rank test p=0.55) and a severe seizure with tonic hindlimb extension (**B**) (Kaplan-Meier curve, Log-rank test p=0.66). **C.** Chemo-convulsant challenge with loxapine (Kaplan-Meier curve, p=0.34, Log-rank test). **D.** Susceptibility to thermogenic seizures (Kaplan-Meier curve, p=0.88, Log-rank test). **E.** The three-chamber social interaction test was used to examine sociability of the mouse model. No significant difference was found in the time spent with the intruder mouse across the genotypes (p=0.207, Kruskal-Wallis test, +/+ n=8, L/+ n=11, L/L n=16). **F.** Spatial memory was not affected in the Y maze test. No differences were found on the time exploring a novel arm in the Y-maze (p=0.261, Kruskal-Wallis test with Dunn's post hoc analysis; +/+ n= 8; L/+ n=8; L/L n=10).

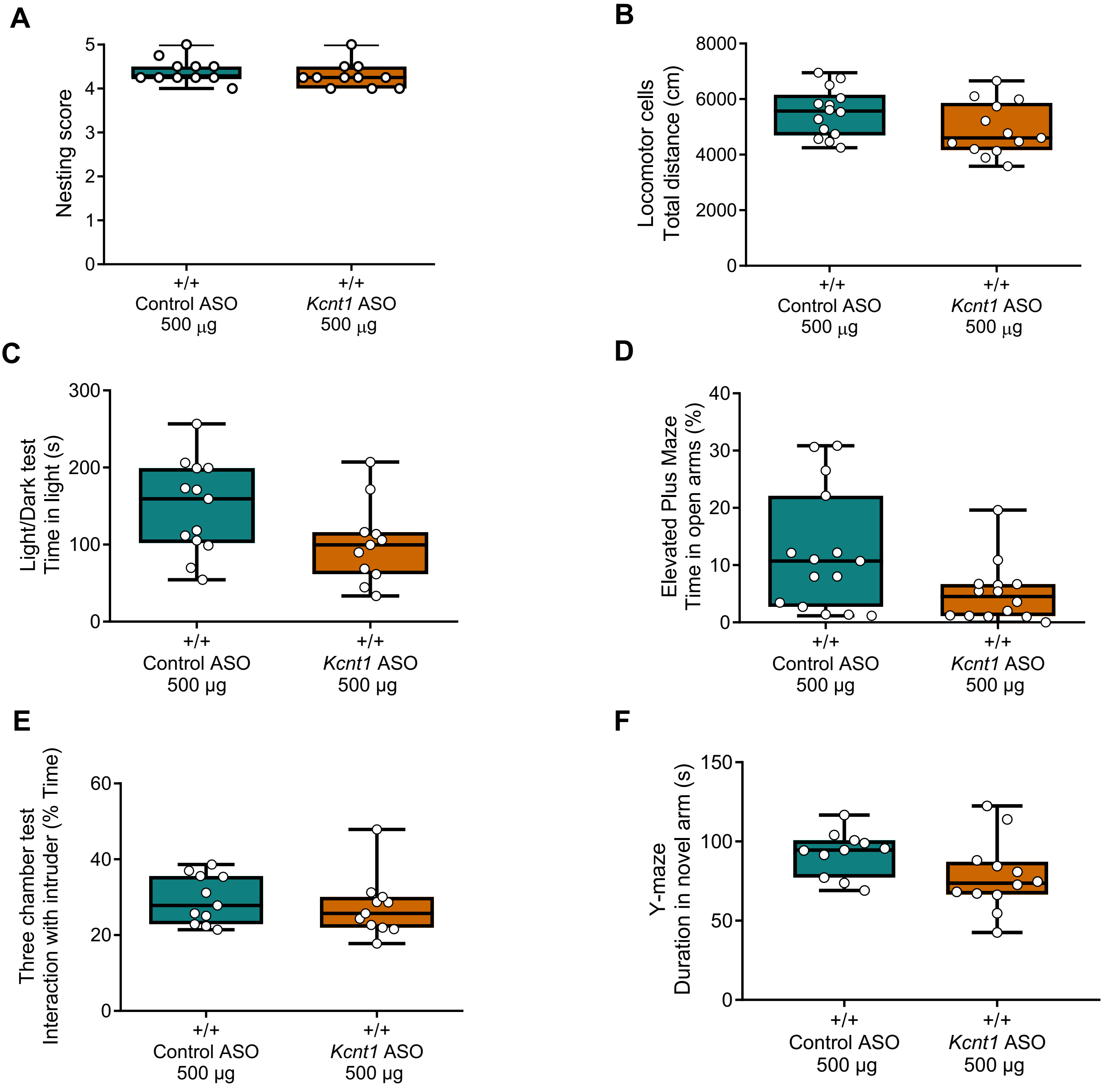

Supplemental Figure 2. Effect of *Kcnt1* ASO administration in adult +/+ mice

A. Nesting behavior was not altered by *Kcnt1* ASO administration (Mann-Whitney test p=0.3136, n=11 for each group). **B.** Behavior in the locomotor cells test was not significantly affected by treatment with *Kcnt1* ASO (Mann-Whitney test p=0.0945, control ASO n=14, *Kcnt1* ASO n=13). **C.** Time spent in the light compartment was reduced although not significantly (Mann-Whitney test p=0.0821, control ASO n=13, *Kcnt1* ASO n=11). **D.** +/+ mice treated with *Kcnt1* ASO showed a reduced time in the open arms compared to control treated animals (p=0.023, Mann-Whitney test, control ASO n=15; *Kcnt1* ASO n=14). **E.** The three-chamber social interaction test showed no difference in the time spent with a stranger mouse (p=0.40,1 Mann Whitney test, control ASO n=11, *Kcnt1* ASO n=11). **F.** Time spent in the novel arm of the Y maze. +/+ mice treated with *Kcnt1* ASO spent less time in the novel arm compared to control treated mice (p=0.0439, Mann Whitney test; control ASO n=11, *Kcnt1* ASO n=12).

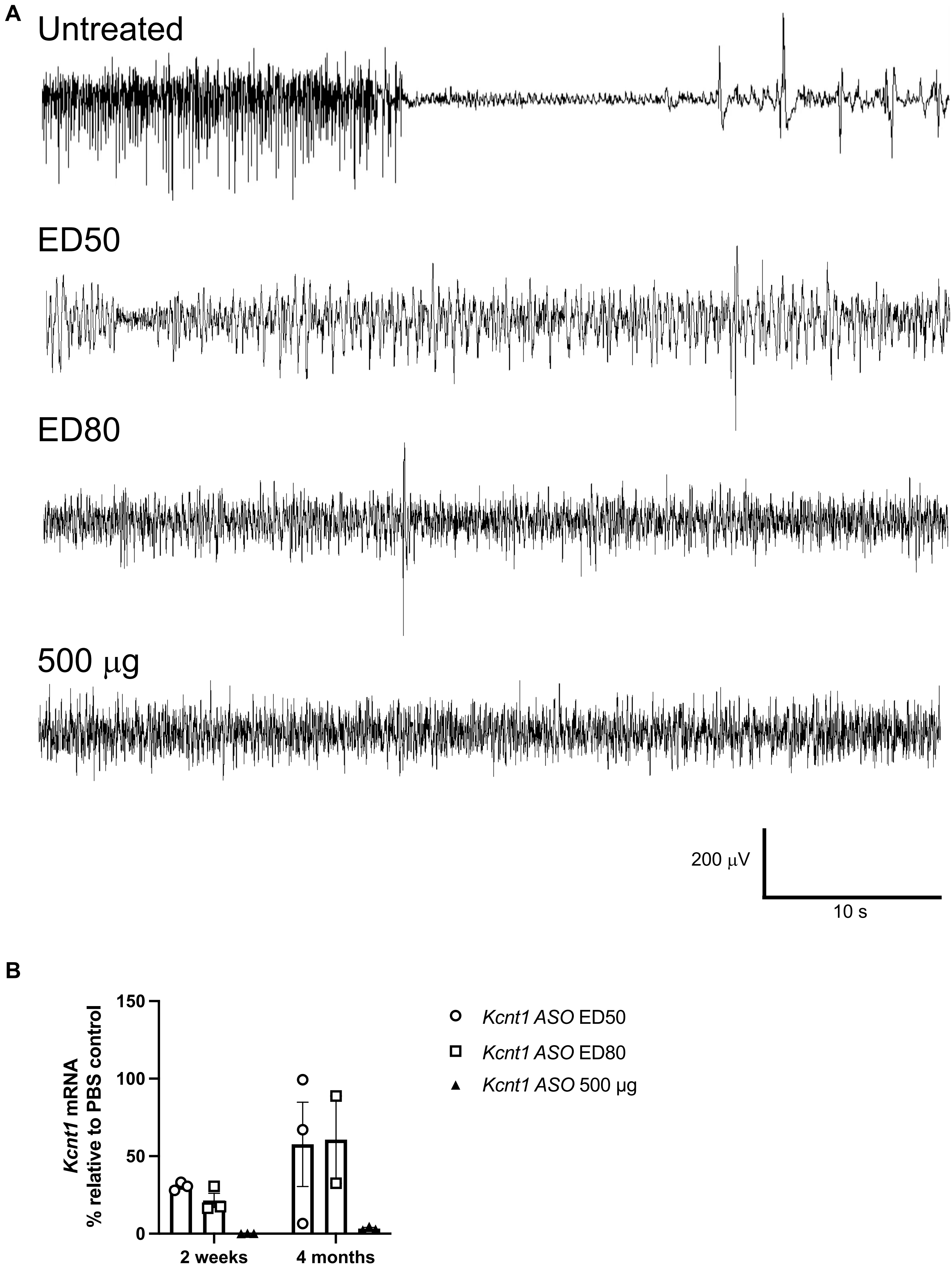

Supplemental Figure 3. A. Representative traces of ECoG signal of mice treated with *Kcnt1* ASO and untreated control. **B.** *Kcnt1* mRNA expression 2 weeks and 4 months after *Kcnt1* ASO i.c.v. injection (n=3-4).

Genotype	L/L	L/+	+/+
Expected	25%	50%	25%
Observed pups (P8-12)	8.8% (n= 23)	61.7% (n=161)	29.5% (n=77)

Supplemental Table 1. Proportion of L/L offspring born from heterozygous crossing. The L/L genotype was found less frequently than expected for a L/+ intercross ($p<0.0001$, Chi-square test; n=40 litters, 261 pups, average litter size of 7 pups).
